# Identification of intima-to-media signals for flow-induced vascular remodeling using correlative gene expression analysis

**DOI:** 10.1038/s41598-021-95403-x

**Published:** 2021-08-09

**Authors:** John Kolega, Kerry E. Poppenberg, Hee-Woong Lim, Liza C. Gutierrez, Sricharan S. Veeturi, Adnan H. Siddiqui, Hamidreza Rajabzadeh-Oghaz, Vincent M. Tutino

**Affiliations:** 1 Canon Stroke and Vascular Research Center, University at Buffalo, Buffalo, NY USA; 2 Department of Pathology and Anatomical Sciences, University at Buffalo, 955 Main Street, Room 4102, Buffalo, NY 14203 USA; 3 Department of Neurosurgery, University at Buffalo, Buffalo, NY USA; 4 Division of Biomedical Informatics, Department of Pediatrics, Cincinnati Children’s Hospital Medical Center, University of Cincinnati College of Medicine, Cincinnati, OH USA; 5 Department of Mechanical and Aerospace Engineering, University at Buffalo, Buffalo, NY USA

**Keywords:** Experimental models of disease, Extracellular signalling molecules, Mechanisms of disease, Aneurysm, Blood flow, Cellular signalling networks, Gene ontology

## Abstract

Changes in blood flow can induce arterial remodeling. Intimal cells sense flow and send signals to the media to initiate remodeling. However, the nature of such intima-media signaling is not fully understood. To identify potential signals, New Zealand white rabbits underwent bilateral carotid ligation to increase flow in the basilar artery or sham surgery (n = 2 ligated, n = 2 sham). Flow was measured by transcranial Doppler ultrasonography, vessel geometry was determined by 3D angiography, and hemodynamics were quantified by computational fluid dynamics. 24 h post-surgery, the basilar artery and terminus were embedded for sectioning. Intima and media were separately microdissected from the sections, and whole transcriptomes were obtained by RNA-seq. Correlation analysis of expression across all possible intima-media gene pairs revealed potential remodeling signals. Carotid ligation increased flow in the basilar artery and terminus and caused differential expression of 194 intimal genes and 529 medial genes. 29,777 intima-media gene pairs exhibited correlated expression. 18 intimal genes had > 200 medial correlates and coded for extracellular products. Gene ontology of the medial correlates showed enrichment of organonitrogen metabolism, leukocyte activation/immune response, and secretion/exocytosis processes. This demonstrates correlative expression analysis of intimal and medial genes can reveal novel signals that may regulate flow-induced arterial remodeling.

## Introduction

Blood flow exerts fluid shear stress on the endothelial lining of arteries. When flow changes, the associated changes in shear stress elicit endothelial responses that alter the vessel wall to accommodate the new flow^1–3^. Although it is endothelial cells (ECs) that directly experience the shear stress, most of the changes in vessel architecture result from activities of the underlying vascular smooth muscle cells (VSMCs). During transient changes in flow, such as the increase that occurs during exercise, ECs respond to the altered shear stress within seconds^4^, sending signals that modulate VSMC contractility to produce vasodilation. The vessel enlarges, but the change is temporary and subsides over the course of several minutes to ~ 2 h when the stimulus is removed^5^. In contrast, sustained changes in shear stress cause long-term structural remodeling of the vessel wall involving changes in cell number and matrix architecture. Flow-sensitive EC signals that regulate VSMC contractility are well characterized, but long-term remodeling appears to be mediated by different mechanisms^6^ and is less understood.

Remodeling requires degradation and synthesis of extracellular matrix (ECM), and thus local production of matrix metalloproteases^7,8^. In some vessel expansion, at least a portion of this matrix turnover can be attributed to inflammatory cells, such as macrophages, invading the vessel wall in response to cytokines and adhesion molecules produced by ECs^9,10^ Inflammatory infiltrates contribute to the degradation and synthesis of extracellular matrix (ECM) and secrete factors promoting growth, proliferation, and/or apoptosis of VMSCs^11,12^. However, removal of endothelium, which would give circulating macrophages direct access to the media, inhibits remodeling both in the case of expansive remodeling under chronic high flow (in response to arteriovenous fistula^13^), and when vessels reduce their diameter under chronic low flow (after partial occlusion^14^). Furthermore, we have shown that when flow is elevated in rabbit basilar arteries (BAs), VSMCs upregulate inflammatory markers and metalloproteases, even before any infiltrating cell populations can be detected^15,16^. These observations suggest that EC signals can directly trigger flow-induced remodeling behavior in resident VSMCs.

Studies have shown that shear stress modulates EC expression of many diffusible signals, including VEGF^17^, PDGF^18,19^, TGF-β^20^, a variety of cytokines^21^, and nitric oxide^22,23^, each of which can stimulate remodeling-related behaviors in VSMCs, such as migration, proliferation, and extracellular matrix production. PDGF expression correlates with constrictive remodeling in response to low flow^24^; genetic knockout of TGF-β receptor in VSMCs inhibited constrictive remodeling in mouse carotid arteries^25^; and both growth factors contribute to recruitment and maturation of mural cells during vessel development^26^. Nitric oxide has been strongly implicated in stimulating arterial enlargement, with several groups showing that disruption of its production prevents or diminishes expansive remodeling under elevated flow^27–31^. However, despite the existing catalog of pathways for EC-to-VSMC communication, the specific molecular events that link stimulation of the intima by flow to structure-altering responses in the media remain sparsely characterized.

In the present study, we demonstrate a strategy for identifying potential molecular signals between endothelium and smooth muscle during flow-induced arterial remodeling. Specifically, we performed transcriptome profiling in the intima and media of rabbit BAs subjected to various flow conditions, and then looked for parallel changes in intimal and medial gene expression that are consistent with a signal-response relationship. We found multiple intimal genes that encode transmittable signals and whose expression correlated with flow-induced changes in medial genes that are involved in arterial remodeling. This correlative approach identified novel potential avenues for crosstalk between ECs and VSMCs related to flow-induced arterial remodeling. The results suggest that vessel structure may be regulated by multiple flow-sensitive signals, and demonstrate that correlative analysis of intimal and medial gene expression is a powerful tool for further dissection of the complex control of arterial structure.

## Results

### Flow environments

**Figure 1:**
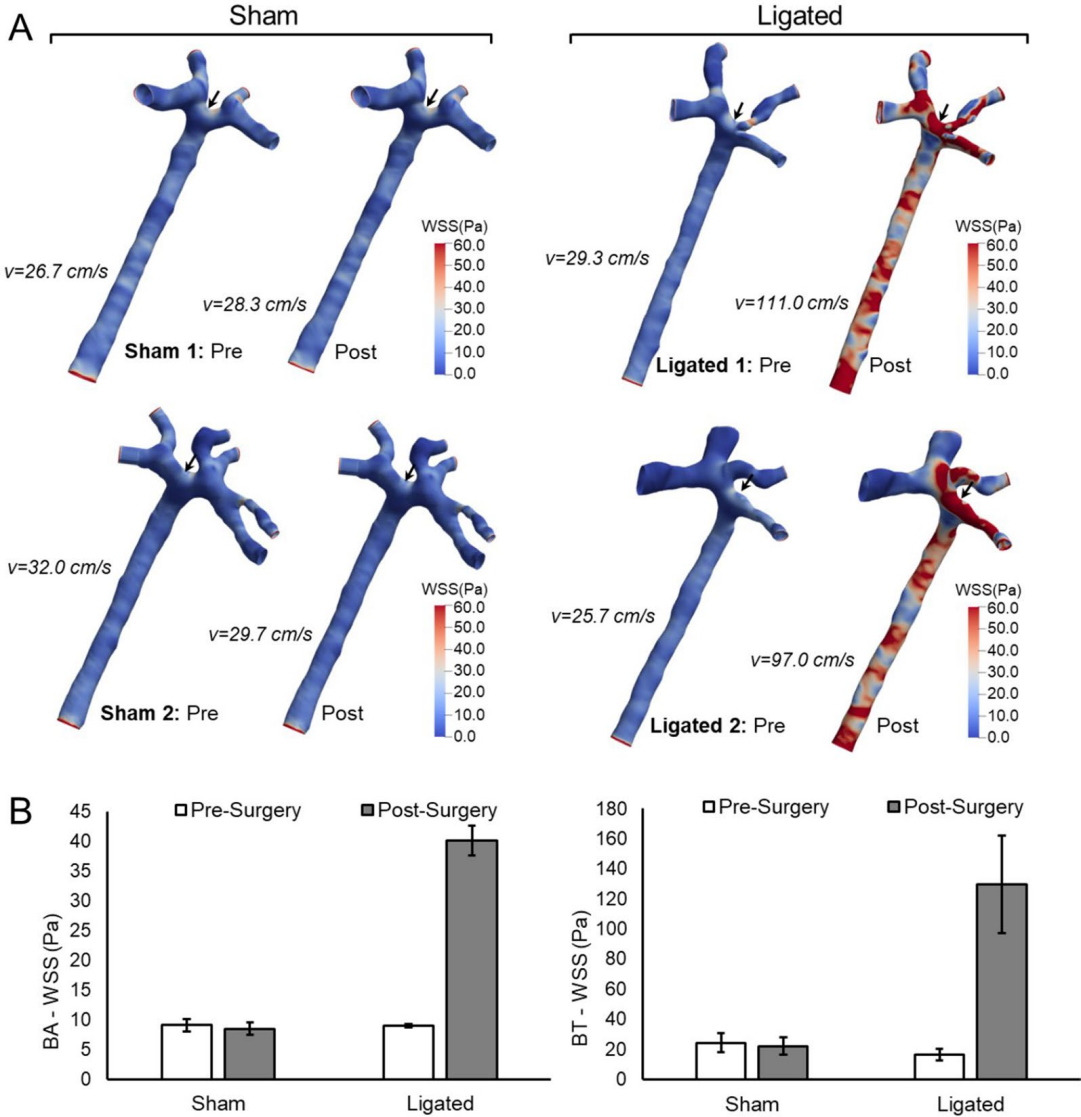
WSS at the BA and BT before and after carotid ligation. (**A**) 3D renderings showing the distribution of WSS at the luminal surface of the BA and BT. WSS was calculated by CFD as described in Materials and Methods based on the indicated flow rates, which were measured by TCD sonography. (**B**) Average WSS values are shown for each location and condition. WSS in the BA was averaged over the length of the vessel, the value for the BT was averaged over the vessel surface within a 1 mm diameter sphere centered at the apex of the bifurcation (arrow). WSS is higher at the BT than the BA, and is increased at both locations after ligation, while sham surgery had no effect.

Flow in the basilar arteries (BAs) of rabbits was modulated by surgically ligating both common carotid arteries for 24 h^4,5,53^. This resulted in a sustained increase in flow in the BA, as determined by transcranial Doppler (TCD) measurements (Supplemental Fig. 1). Wall shear stress (WSS) in the BA and at the basilar terminus (BT) was computed from the flow velocity and vessel geometry (determined by 3D angiography) using computational fluid dynamics (CFD). Figure 1A,B show that WSS is slightly higher at the BT than in the BA, and that the increased flow after ligation was accompanied by increased WSS throughout the BA (average WSS = 40 Pa in ligated rabbits vs. 10 Pa pre-ligation or in sham animals) and an even larger increase at the BT (from ~ 20 Pa in unligated animals to ~ 130 Pa after ligation). We also calculated oscillating flow index, cross flow index, WSS gradient in the direction of flow and transverse WSS at each location (Supplemental Fig. 2). Ligation produced no consistent changes in oscillating flow index or cross flow index, while both longitudinal and transverse gradients in WSS tended to increase in parallel with higher WSS, as would be expected.

### Separation of intimal and medial tissue from discrete flow environments

Tissue was harvested for analysis of gene expression 24 h after ligation, which is well past the window for activating vasodilation or constriction. Apoptosis, matrix degradation, and medial thinning are evident by 48 h after ligation^15^, so gene expression persisting after 24 h represents sustained responses that may contribute to long-term remodeling. In order to examine gene expression in ECs and VSMCs at locations subjected to different WSS, we performed RNA-seq on intima and media from four different flow environments: (1) the straight portion of the BA in animals with normal flow, (2) the regions flanking the apex of the BT in animals with normal flow, where WSS is slightly higher than in the BA, (3) the BA after carotid ligation, where WSS is elevated, and (4) the BT after ligation, where the highest levels of WSS were found (Fig. 1). The tissues were obtained by cutting frozen sections of the arteries and using laser microdissection to collect the desired regions. BA tissue was collected in longitudinal strips distributed over the full length of the straight portion of the artery proximal to the BT, and BT tissue was collected from the wall flanking the bifurcation apex, extending 400–500 µm downstream into the branches on each side (Supplemental Fig. 3). The dissected materials correspond to the regions over which WSS is averaged for the graphs in Fig. 1B, and for later comparisons of gene expression relative to local WSS.

**Figure 2:**
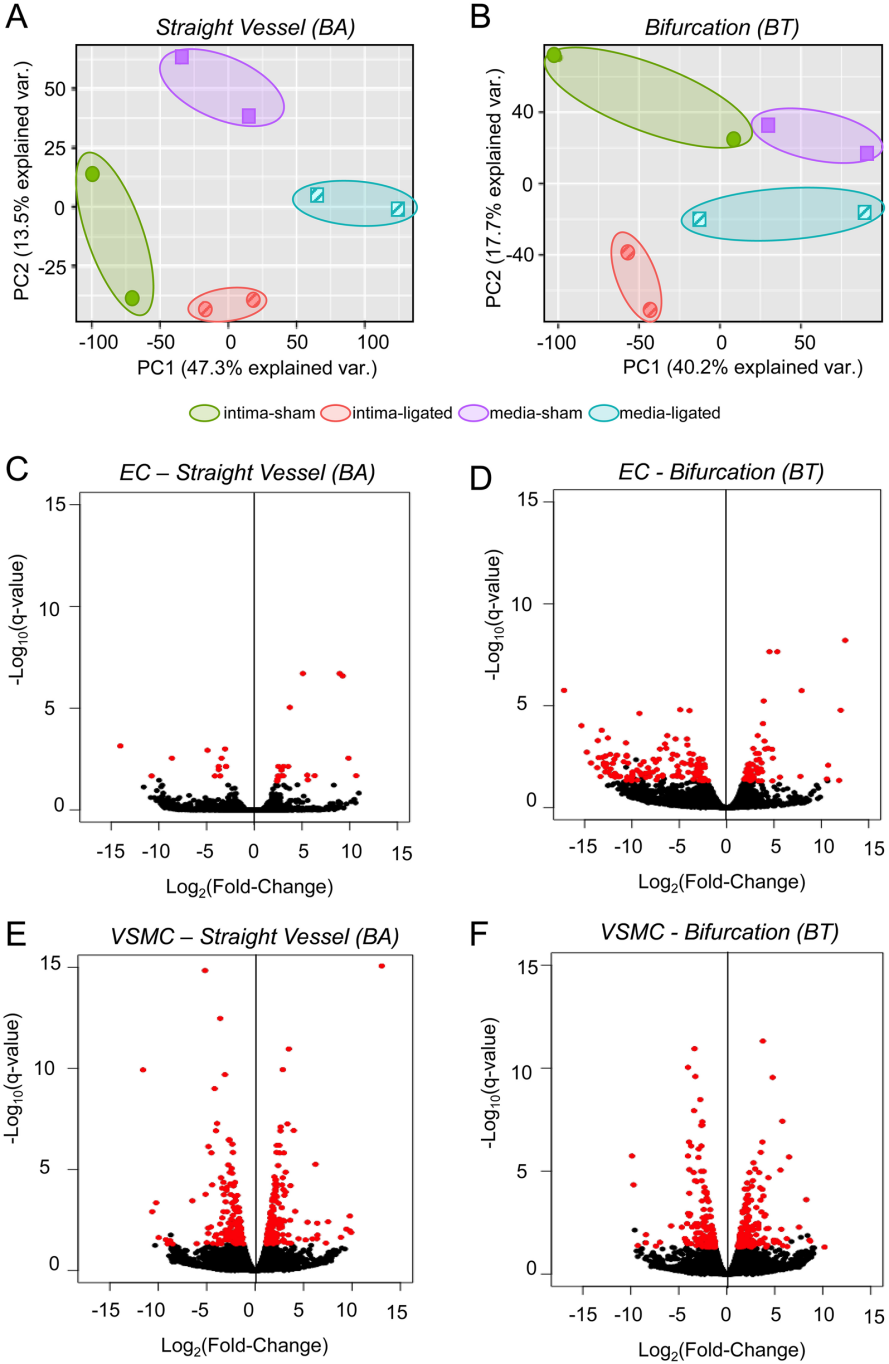
Principal component analysis of gene expression levels in the 16 samples, and differential expression analysis. (**A**, **B**). Principal component analysis was performed on the gene expression levels for all genes that had a count sum > 0 across all samples. The first two principal components for the collective gene expression in each sample are plotted for samples from (**A**) straight vessel segments and (**B**) bifurcations. In both cases, intima samples (circles) are spatially separated form media samples (squares). In addition, sham tissues (solid symbols) are distinguished from tissues taken from ligated animals (hatched symbols). Colored ellipses indicate paired duplicates from 2 different rabbits. (**C**–**F**) Gene expression in tissues from ligated and unligated rabbits was compared by RNA-seq. Volcano plots show fold change and p-value for comparisons at BA intima (**C**), BT intima (**D**), BA media (**E**), and BT media (**F**). Each dot represents one gene, and red dots indicate DEGs, i.e. detectable genes with q-value < 0.05.

Note that laser microdissection allowed us to obtain separate samples from the intima and from the media at each location. Thus, 16 samples were obtained: from the intima and media of the BA and BT of two rabbits with ligated carotid arteries and from the same locations in two un-ligated rabbits. cDNA libraries were then constructed from each sample. Based on RNA-seq performed at a depth of > 5 × 50 reads, we found that in each sample > 70% of reads aligned to exons. Expression of the endothelial markers, *PECAM1*, *VWF*, and *CDH*5 was minimal or undetectable in medial samples and abundant in the intimal samples. *PECAM1* expression was on average 34-fold higher in the intima; *VWF* expression 12-fold higher, and *CHD5* at least 7-fold higher (*CHD5* was undetectable in two media samples and so “infinitely more abundant” in the corresponding intima). Because the intima is very thin and extremely close to the media, it was impossible to collect the full thickness of the endothelial layer without obtaining some smooth muscle during laser microdissection. Thus, there was a greater proportion of medial “contamination” in the intimal samples. In the intima, average levels of the smooth-muscle markers, *DES*, *ACTA2*, and *MYH11,* were 18%, 37%, and 43% of the levels in the media, respectively. Relative expression of the endothelial and smooth-muscle markers for each pair of intima-media tissues are shown in Supplemental Fig. 4. Principal component analysis of gene expression levels in the 16 samples showed separation of intimal from medial samples at both locations, as well as separation between samples exposed to normal versus high flow (Fig. 2A,B; Supplemental Fig. 5).

We also found no detectable expression of the genes encoding CD14, 16, 19, 64 or 68, cell surface markers found on monocyte, macrophages, and B- and T-cells in the transcriptomes of ligated or unligated animals. This supports our previous reports that there is little or no infiltration of the artery wall by inflammatory cells during the early stages of flow-induced remodeling^15,16^, and is consistent with endothelial and smooth muscle cells being the dominant contributors to our intimal and medial transcriptomes.

### Differential gene expression in the intima under high flow

Comparison of RNA-seq data for tissues from ligated versus unligated animals revealed numerous genes in both the intima and media whose expression was detectable, i.e. genes with maximum average expression > 1 reads per kilobase of transcript per million mapped reads (RPKM) in each group. We defined differentially expressed genes (DEGs) as those that had a false discovery rate corrected p-value (q-value) < 0.05 for any comparison (Fig. 2C–F). In the intima, there were 31 DEGs in the straight BA segment (19 with increased expression, 12 with decreased expression) and 180 DEGs at the BT bifurcation (54 with increased expression, 126 with decreased expression) (Fig. 2C,D). These include 21 genes that were differentially expressed at both locations.

Examination of the gene ontologies associated with these expression changes revealed patterns consistent with production of signals for transmission to VSMCs in the media (Supplemental Table 1). Intimal DEGs that were upregulated in straight vessels after ligation were enriched in terms associated with hormone regulation, secretion/export, and hydrogen peroxide production. Hydrogen peroxide is a known vasoregulator of cerebral and mesenteric arteries^32–35^. Hormone regulation was also the most significant biological process returned by gene ontology of upregulated genes at the BT. Upregulated intimal genes at the BT were additionally associated with inflammatory response, regulation of fluid and coagulation, and numerous processes related to cell movement, possibly reflecting a wound-type response. Meanwhile, DEGs that were downregulated by increased flow were enriched for keratan sulfate catabolism in the intima of straight-vessels, and, in the intima at bifurcations, downregulated DEGs were enriched for biological processes involved in regulation of hydrolase and peptidase activities and chemotaxis, again suggesting a wound-like phenotype.

### Differential gene expression in the media under high flow

Comparison of gene expression in the media of ligated versus unligated animals revealed 334 genes that were differentially expressed in the BA (190 with increased expression, 144 with decreased expression) and 303 genes at the BT (159 with increased expression, 144 with decreased expression) (Fig. 2E,F). 113 genes were differentially expressed at both locations. Gene ontologies of the DEGs in the media were indicative of extensive nuclear re-organization as well as re-structuring at the whole-cell level (Supplemental Table 2). In straight vessel segments, DEGs that were upregulated by increased flow were associated with biological processes related to DNA synthesis and telomere maintenance, and RNA/protein localization in Cajal bodies and the nucleus, reflecting altered nuclear structure. Biological processes involved in cell and tissue rearrangement were also overrepresented, including multiple terms for extracellular matrix and integrin signaling; development and remodeling; and cell migration and localization. Finally, the ontologies returned significant terms for leukocyte activation, cytokines, and responses to stress and chemical/organic substances, possibly reflecting pathways that recruit other local and systemic cells for remodeling responses. Significant terms associated with DEGs that had decreased expression were related to ECM organization and cell adhesion.

At the BT bifurcation, flow-increased DEGs in the media were associated with wound healing, locomotion, and remodeling, and with immune cell activity and responses to chemical and organic substances, as was found in straight segments. In addition, coagulation, hemostasis and secretion/exocytosis were overrepresented biological processes at the BT, consistent with extracellular remodeling events. Terms associated with down-regulated DEGs included matrix/structure organization, hydrolase activity, adhesion, and muscle development. Such changes may reflect VSMCs de-differentiating from the so-called contractile state to a more synthetic phenotype, which occurs during vascular remodeling^15,16,36^.

### Potential intimal signals for medial remodeling

When flow increases in a vessel, ECs in the intima are exposed to increased WSS. In order for this to elicit remodeling behaviors by the VSMCs in the media, ECs presumably express and release transmittable signal(s). Of the 194 flow-modulated intimal DEGs, 49 code for extracellular products (Table 1). The expression of 10 of these extracellularly-expressed DEGs were significantly affected by increased flow in straight vessels (3 were upregulated and 7 were downregulated), and 44 exhibited significant changes at bifurcations (11 upregulated and 33 downregulated). To examine whether the products encoded by these intimal genes were likely to be affecting VSMCs in the media, we looked for correlations between intimal and medial gene expression.

**Table 1:** Intimal DEGs encoding extracellular products.

DEG	Product class	Bifurcation		Straight vessel	
		Log2(F-C)	q-value	Log2(F-C)	q-value
**Up-regulated genes**					
*CHI3L2*	Enzyme	11.88	0.0469^a^	0.00	1.0000
*SERPINA6*	Other	10.72	0.0084^a^	5.06	0.4320
*HBEGF*	GF	4.80	0.0014^a^	1.08	1.0000
*TIMP1*	Cytokine	4.52	0.0000^a^	3.74	0.0000^a^
*ADAMTS9*	Peptidase	4.08	0.0013^a^	2.70	0.1782
*SERPINE1*	Other	3.91	0.0000^a^	2.49	0.0216^a^
*SEMA3F*	Other	3.39	0.0022^a^	2.12	0.3327
*BMP2*	GF	3.35	0.0497^a^	0.34	1.0000
*IL15*	Cytokine	3.11	0.0142^a^	1.79	0.6464
*PLAT*	Peptidase	2.77	0.0261^a^	1.35	0.8802
*ADAMTS6*	Peptidase	2.63	0.0456^a^	1.74	0.6622
*GLIPR1*	Other	1.06	0.5043	2.42	0.0340^a^
**Down-regulated genes**					
*PTN*	Growth factor	− 17.17	0.0000^a^	− 7.86	0.2483
*PROS1*	Other	− 14.76	0.0019^a^	− 1.58	1.0000
*MFAP5*	Other	− 12.86	0.0030^a^	− 9.54	0.1498
*VIT*	Other	− 12.80	0.0030^a^	1.44	1.0000
*CST6*	Other	− 12.76	0.0107^a^	− 8.97	0.3702
*C1QA*	Other	− 12.48	0.0186^a^	− 0.78	1.0000
*TTR*	Transporter	− 12.18	0.0275^a^	− 7.27	0.7186
*C2*	Peptidase	− 11.60	0.0265^a^	− 2.14	1.0000
*CXCL10*	Cytokine	− 11.55	0.0027^a^	3.42	0.7338
*NID2*	Other	− 10.86	0.0056^a^	− 0.75	1.0000
*C1QTNF7*	Other	− 10.64	0.0007^a^	− 8.63	0.0029^a^
*CXCL11*	Cytokine	− 10.15	0.0322^a^	− 0.88	1.0000
*BMP3*	Growth factor	− 9.57	0.0045^a^	− 0.12	1.0000
*HP*	Peptidase	− 9.10	0.0080^a^	− 1.54	1.0000
*C1QC*	Other	− 8.76	0.0056^a^	− 1.71	1.0000
*ITIH2*	Other	− 8.61	0.0206^a^	1.24	1.0000
*IGF1*	Growth factor	− 8.54	0.0339^a^	0.80	1.0000
*PLA2G7*	Enzyme	− 8.30	0.0112^a^	− 3.68	0.7504
*LYPD6*	Other	− 7.78	0.0182^a^	− 9.65	0.0609
*RSPO3*	Kinase	− 6.86	0.0664	5.41	0.9693
*ANGPTL4*	Other	− 6.76	0.0253^a^	− 2.53	0.9693
*NDP*	Growth factor	− 6.71	0.0583	− 10.76	0.0216^a^
*SPP1*	Cytokine	− 5.39	0.0004^a^	− 4.13	0.0216^a^
*IGFBP2*	Other	− 5.12	0.0286^a^	− 2.92	0.6982
*OGN*	Growth factor	− 4.91	0.0000^a^	− 2.19	0.3549
*SERPINF1*	Other	− 4.70	0.0322^a^	1.87	0.9852
*CYTL1*	Cytokine	− 4.14	0.0008^a^	− 3.74	0.0073^a^
*LUM*	Other	− 4.07	0.2310	− 14.04	0.0007^a^
*OMD*	Other	− 3.91	0.0000^a^	− 2.94	0.0073^a^
*ISG15*	Other	− 3.56	0.0024^a^	0.01	1.0000
*LCAT*	Enzyme	− 3.52	0.0417^a^	− 1.14	1.0000
*ABI3BP*	Other	− 3.21	0.0196^a^	− 3.57	0.0216^a^
*CYR61*	Other	− 3.12	0.0126^a^	− 1.61	0.7504
*SELENOP*	Other	− 2.83	0.0040^a^	− 1.84	0.3549
*COL14A1*	Other	− 2.82	0.0117^a^	− 0.79	1.0000
*IGFBP6*	Other	− 2.81	0.0042^a^	− 0.52	1.000
*SAMD9L*	Other	− 2.41	0.0483^a^	0.92	1.000

All genes whose expression was significantly different between ligated and unligated animals in the intima of bifurcations (BT) or straight vessels (BA) and whose product was classified as extracellular in the Ingenuity Pathway Analysis database are listed.

*DEG* differentially expressed gene, *F-C* fold-change.

^a^Significantly different expression between ligated and unligated.

For each intimal DEG, we compared its expression across the eight sample locations (BA and BT in two ligated and two unligated animals) with the expression of each individual medial DEG at the same location. We found 14,286 positively correlating gene pairs (Pearson correlation coefficient > 0.9; p < 0.0001), and 15,491 gene pairs that correlated negatively (Pearson correlation coefficient < − 0.9; p < 0.0001). For each of the 194 intimal DEGs, expression levels correlated with between 6 and 301 different genes in the media (Supplemental Table 3). The 49 intimal DEGs that encode extracellular products correlated with 6 to 287 medial genes, with 18 extracellular-encoding intimal DEGs having > 200 medial correlates (Table 2). We focused further analysis on these 18 highly correlated intimal DEGs.

**Table 2:** Highly correlated intimal DEGs with extracellular products.

Intimal gene	# of medial correlates	# of positive correlates	# of negative correlates	Gene product
**Up-regulated by flow**				
*SERPINA6*	287	182	105	Serpin A6
*GLIPR1*	257	153	104	Glioma pathogenesis-related protein 1
*SERPINE1*	255	152	103	Serpin E1
*ADAMTS9*	235	132	103	A disintegrin and metalloprotease with thrombospondin motifs 9
*TIMP1*	224	123	101	Tissue inhibitor of metalloprotease 1
*IL15*	219	112	97	Interleukin 15
**Down-regulated by flow**				
*CYTL1*	283	106	177	Cytokine-like protein 1
*NDP*	281	104	171	Norrin
*SPP1*	269	89	180	Osteopontin
*C1QTNF7*	267	104	163	C1q and tumor necrosis factor-related protein 7
*LYPD6*	254	84	170	Ly6/PLAUR domain-containing 6
*LUM*	251	95	156	Lumican
*CYR61*	250	93	157	Cell communication network factor 1
*ABI3BP*	239	95	144	ABI family member 3 binding protein
*CST6*	233	74	159	Cystatin E/M
*PLA2G7*	215	76	139	Phospholipase A2 group VII
*OMD*	204	100	104	Osteomodulin
*MFAP5*	202	61	141	Microfibril associated protein 5

For each Intimal DEG encoding an extracellular product, the number of medial DEGs whose expression significantly correlated was determined. The intimal DEGs with > 200 medial correlates are listed. The upper portion of the table shows genes that were upregulated by increased flow (at both the BA and BT) and the bottom genes were decreased by flow (at both the BA and BT).

### Gene ontologies of medial genes that correlate with flow-sensitive intimal genes

**Figure 3:**
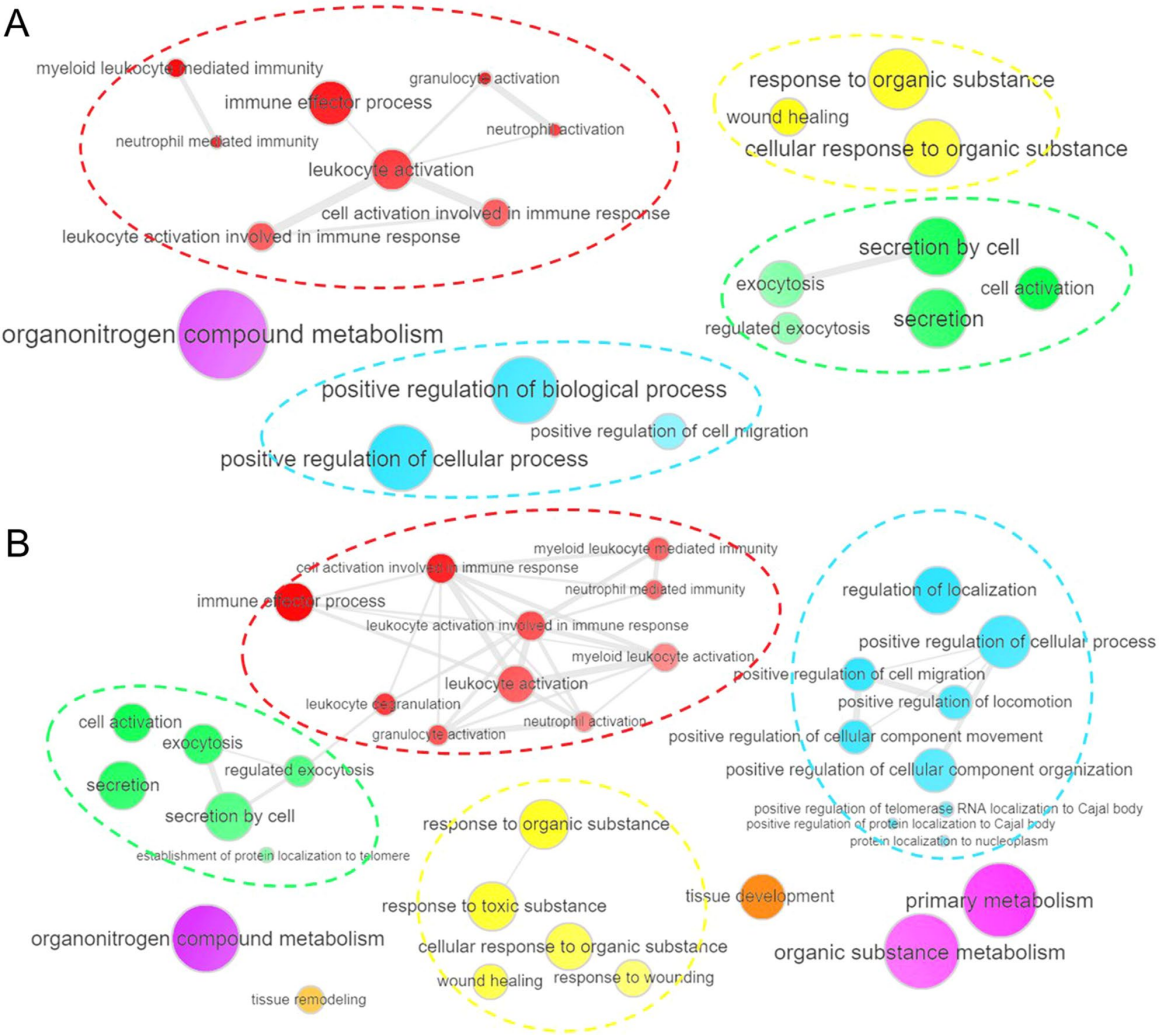
Biological processes associated with medial correlates of flow-sensitive intimal genes. For all the medial genes whose expression correlated with the 18 flow-sensitive, extracellular-coding intimal genes that had > 200 medial correlates, significantly (q-value < 0.05) over-represented GOs were identified using g:Profiler. The diagrams summarize the biological processes terms that were present in > 50% of GOs enriched in (**A**) genes positively correlated with intimal genes exhibiting higher expression in ligated animals compared to sham, and (**B**) genes negatively correlated with intimal genes exhibiting lower expression in ligated animals than in sham. Symbol sizes represent the frequency of the ontology term, and lines between symbols indicate relationships. Groups of closely related terms are denoted by matching color and dashed ellipses. Note that organonitrogen compound metabolism is the largest symbol in (**A**), and one of the largest in (**B**). Both diagrams show multiple terms associated with leukocyte activation/immune responses (red) and secretion/exocytosis (green). Terms related to wound healing and cell movement are also present in both summaries.

To determine what responses the 18 most-correlated, extracellular product-encoding, intimal DEGs might signal in VSMCs, we performed gene ontology enrichment analysis on the correlating medial genes. Six of the 18 genes of interest exhibited increased expression in response to ligation both in bifurcations and in straight vessels, and the remaining 12 had decreased expression at both sites. Although all six upregulated genes displayed higher expression at both locations in ligated animals, in the BA the difference was statistically significant only for GLIPR, TIMP1, and SERPINE1 (all except *GLIPR1* were significantly upregulated by flow at the BT). This may reflect the much higher post-ligation WSS experienced at the BT. For each of the six upregulated genes, we examined gene ontologies of the positively correlating medial genes in g:Profiler. All significantly enriched ontologies (q-value < 0.05) from the six gene sets were pooled and ranked by occurrence, and the rankings were used to reduce and summarize the biological process gene ontologies to facilitate identification of the most important regulated ontologies. As illustrated in Fig. 3A, the most prominent biological process was organonitrogen compound metabolism, consistent with previous reports of an important role for nitrous oxide in regulating arteriogenesis^27–31^. In addition, two groups of multiple terms were related to leukocyte activation/immune responses (red ontologies in Fig. 3A) and secretion/exocytosis (shown in green in Fig. 3A), consistent with the pro-inflammatory environment and accompanying release of cytokines, proteases, and matrix proteins that occur during vascular remodeling^9,10,37–40^. Wound healing and positive regulation of cell migration were also significantly associated with the up-regulated gene sets. The same analysis applied to the negatively correlating medial genes did not yield any significantly enriched biological processes.

Analysis of the 12 highly correlating intimal genes (> 200 medial correlates) that were down-regulated by increased flow revealed similar medial responses. Again, most genes changed significantly at the BT, where WSS was higher (all but *NDP* and *LUM* were significantly lower in ligated animals), and fewer genes at the BA exhibited differences that were statistically significant (*NDP, LUM, C1QTNF7, SPP1, CYTL1,* and *OMD* were lower post-ligation, but q values were > 0.05 when comparing ligated and unligated animals). The medial genes that were positively correlated with the 12 down-regulated intimal genes (i.e., medial genes that would also be down-regulated under increased flow), did not yield significant gene ontologies. However, the negatively correlating genes (which are up-regulated under increased flow) were associated with organonitrogen compound metabolism (Fig. 3B), leukocyte activation/immune responses (shown in red) and secretion/exocytosis (in green) like the positive correlates of upregulated intimal genes. In addition, terms related to cell migration and wound healing, and tissue remodeling and tissue development, were among the significant biological processes associated with negative correlates. Thus, down-regulation of the respective intimal genes is predicted to produce medial responses that are similar to those elicited by the intimal genes that are up-regulated under the same conditions.

### WSS dose response of gene expression

**Figure 4:**
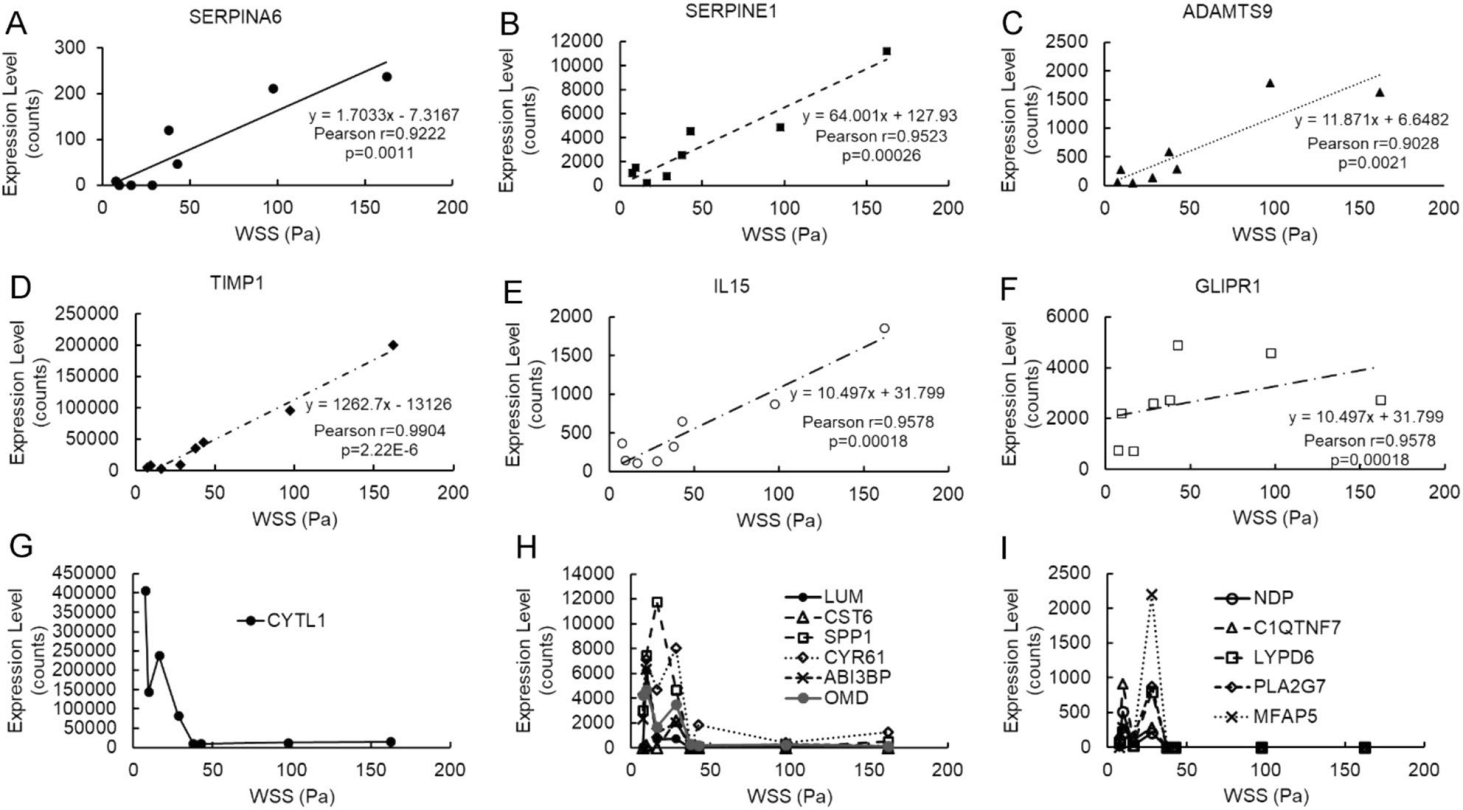
Gene expression as a function of local WSS. The expression levels for the 18 intimal genes with > 200 medial correlates (as determined by RNA-seq) are shown as a function of the WSS at the sample’s location (as determined by CFD). Expression levels of *SERPINE6* (**A**), *SERPINE1* (**B**), *ADAMTS9* (**C**), *TIMP1* (**D**), and *IL15* (**E**) were all positively correlated with WSS over the full range of observed values (Pearson r > 0.9). *GLIPR1* (**F**) increased with increasing WSS, but plateaued at WSS > 40. The remaining genes displayed very non-linear “dose–response” curves, shown in (**G**) *CYTL1*, (**H**) *LUM*, *CST6*, *SSP1*, *CYR61*, *ABI38P* and OMD, and (**I**). *NDP*, *C1QTNF7*, *LYPD6*, *PLA2G7*, and *MFAP5*. The maximal expression values for these 12 genes are all at WSS below 40 Pa, and expression is dramatically lower at WSS > 50 Pa.

To examine more closely the relationship between blood flow and the expression of potential remodeling signals, gene expression levels for the 18 intimal genes with > 200 media correlates were plotted as a function of local WSS. In each animal and at each location for which expression was measured, the local WSS was calculated as the average WSS over the surface from which the tissue was microdissected. The expression levels for 5 of the 18 genes (*ADAMTS9*, *IL15*, *SERPINA6*, *SERPINE1*, and *TIMP1*) had strong positive correlations with average WSS that were well-described by a linear relationship (r > 0.8) (Fig. 4A–E). *GLIPR1* trended similarly, with expression generally higher at higher WSS, but expression plateaued at intermediate WSS levels and the dose–response did not fit a linear curve (Fig. 4F). The remaining 12 genes were down-regulated in response to elevated flow, with markedly non-linear responses (Fig. 4G–I). For each of these 12 genes, the highest levels of expression were observed between ~ 10 and ~ 30 Pa, and minimal or no expression was detected in tissues experiencing WSS > 50 Pa.

## Discussion

These experiments investigated the earliest changes in gene expression during flow-induced arterial remodeling described to date. Moreover, by using laser microdissection to separate the intima and media, the responses of the endothelial layer in the intima, which is the direct sensor of WSS (the mechanical driver of remodeling), are separated from downstream effects on cells in the media, where most of the actual remodeling occurs. This reveals potential signaling mechanisms between the endothelial sensor of flow (i.e. ECs) and the primary effectors of remodeling within the vessel wall (VSMCs) that have not been previously identified.

Flow-induced changes in expression of intimal genes that code for extracellular products represent a possible mechanism for EC signaling to the media. Changes in the expression of signaling genes should elicit changes in the target cells, and by examining gene expression in both the signaling and target tissues across a variety of conditions, we identified over 29,000 gene pairs whose expression levels were significantly positively or negatively correlated. Most notably, 18 of the intimal genes that encode extracellular products each had over 200 correlating genes in the media consistent with a signal-response relationship. Furthermore, several of these 18 genes have previously been implicated as effector molecules in vascular remodeling in mice. For example, the products of *CYR61* (also known as *CCN1*) and *CYTL1* stimulate, and lumican (encoded by *LUM*) inhibits, angiogenesis in tumor, skin pouch, and in vitro assays^41–44^; *NDP* acting via Fz4/Lrp receptors regulates retinal vascularization^45^; and *SSP1* codes for the cytokine osteopontin, which is required for post-ischemic neovascularization in a femoral artery ligation model^46^. In rats, *IL15*, which encodes the pro-inflammatory cytokine interleukin 15, is highly expressed in the ductus arteriosus during development, but its expression declines dramatically after birth when the ductus shrinks and closes^47,48^.

For most of the intimal genes that were upregulated by WSS, expression was proportional to WSS over a wide range of magnitudes. This is consistent with the behavior of signals that function to induce remodeling aimed at reducing WSS; i.e., more severe hemodynamic stress should elicit a stronger signal in order to cause more rapid and extensive remodeling. In contrast, intimal genes that were down-regulated by WSS displayed a dramatic decline in expression when WSS exceeded a range of 10–50 Pa. These down-regulated genes might be involved in maintaining structural homeostasis under normal flow. Loss of maintenance signals that are otherwise constitutively expressed could permit VSMCs to revert to a “dedifferentiated” state that is migratory and pro-inflammatory^11,12^.

The number of flow-sensitive intimal genes that code for extracellular products and whose expression correlates with many medial genes may reflect a complex and sensitive regulatory mechanism for flow-induced remodeling, in which different aspects of medial responses are controlled by different signals. For example, molecules like adamts9, lumican, phospholipase A2, serpins A6 and E1, and tissue inhibitor of metalloprotease (which are encoded by *ADAMTS9*, *LUM*, *PLA2G7*, *SERPINA6*, *SERPINE1*, and *TIMP1*, respectively) may drive remodeling of extracellular matrix, while cytokines like C1qTNF, interleukin 15, norrin, osteomodulin, and osteopontin (encoded by *C1QTNF*, *IL15*, *NDP*, *OMD*, and *SSP1*), may primarily modulate cell proliferation and migration. Alternatively, VSMCs may regulate remodeling behaviors based on integration of multiple signals in order to provide graded responses to different levels of flow stimuli. Such an arrangement would allow more diverse and more nuanced behaviors than binary on–off regulation by one or two signaling molecules. We predict that characteristic signal profiles will be observed for different remodeling scenarios. For example, short-term elevation of flow elicits transient and reversible dilation, whereas sustained high WSS induces tissue growth and matrix restructuring. Thus, the signal profile expressed in ECs may change over time as cells inure to persistent mechanical stimulus. Similarly, high WSS can stimulate robust enlargement of the vessel wall, but in other instances will trigger destructive remodeling that leads to wall thinning and aneurysm formation^47,48^. The molecular basis for such selective initiation of constructive versus destructive remodeling is currently unknown, but it could involve differences in the regulatory signals elicited in the ECs.

Our study has several limitations. First, the small number of biological replicates limits the statistical confidence of gene expression values, and consequently may reduce the number of DEGs identified. Nonetheless, these preliminary data show the technical feasibility of locally correlating intimal and medial gene expression, and demonstrate the power of this approach for generating novel insights into possible regulatory pathways. Second, we only examined RNA expression. It is important that future work determine if the observed changes in RNA expression for putative signaling proteins in the intima are reflected in protein production, and that the protein products reach their expected targets. Similarly, it will be necessary to confirm whether the expression of correlated genes in the media result in protein production. We also plan to investigate whether the medial responses are primarily the result of expression changes in VSMCs, or if other cell types contribute. Third, examination of more animals and additional locations and hemodynamic conditions will be needed to establish which expression changes contribute to particular aspects of the flow response. More diverse hemodynamic conditions will likely provide insight into the specific role of different signals in the remodeling process. For example, studying gene expression during long- versus short-term stimulation may help define the mechanism for the transition between transient dilation and permanent structural remodeling. Likewise, comparison of expression profiles during the onset of constructive vessel expansion versus the beginning of destructive remodeling such as occurs during aneurysm initiation^9,10,15,49^, could identify discriminating signals. Mapping gene expression during aneurysm initiation will likely require more detailed calculation of hemodynamics and higher resolution tissue isolation. This is particularly important near bifurcations, where flow patterns are complex. Some of the differences between BA and BT gene expression may represent responses to other hemodynamic forces. For example, it has been shown that spatial gradients in WSS are an important factor in triggering aneurysm formation^47,48,50^, and gradients superimposed on high WSS elicit different responses from endothelial cells in culture than uniform high WSS of the same magnitudes^51,52^. Spatial gradients in WSS add to the shear force on the luminal surface of endothelial cells and may augment their responses to flow. WSS gradient was higher at the BT after ligation in the present study, as was transverse WSS, which would also add to the mechanical stress experienced by the endothelium. These extra forces should be considered as potential contributors to the mechanical triggers for differential remodeling responses. Lastly, it will be necessary to manipulate putative signaling molecules in order to test whether they are necessary and sufficient for particular remodeling behaviors. Ideally this would be accomplished with conditional mutants of the genes of interest. Given the limited molecular engineering in rabbits, such studies might be better conducted in mice or rats.

In conclusion, flow-dependent arterial remodeling likely involves the induction of multiple intimal genes for signals that elicit remodeling responses in the media. Correlation analysis is a powerful approach for identifying potential signals for such intercellular regulatory communication, and laser microdissection allows examination of that communication with intra-tissue resolution. Our analysis suggests multiple novel signaling molecules and the potential for complex signal-response profiles for regulating diverse arterial remodeling behaviors.

## Methods

### Animal model

Female New Zealand white rabbits (4–5 kg) underwent bilateral common carotid artery ligation surgery (n = 2) or a sham surgery (n = 2), as previously described^53^. Bilateral ligation of the common carotid artery increases flow through the BA and causes destructive remodeling at the BT. Before surgery, all rabbits were imaged by rotational angiography to capture the 3D geometry of the BA and the BT, and all were subjected to TCD ultrasonography to measure blood flow rates in the BA. 24 h after surgery, the rabbits were again subjected to imaging and TCD. They were then euthanized by intravenous injection of 100 mg/kg sodium pentobarbital. All procedures were approved by the Institutional Animal Care and Use Committee of the University at Buffalo. The University at Buffalo’s Laboratory Animal Facility was certified at the highest level by the American Association for the Assessment and Accreditation of Laboratory Animal Care International and met or exceeded the guidelines of the United States Department of Agriculture. Results from this study are reported in accordance with the ARRIVE (Animals in Research: Reporting In Vivo Experiments) guidelines^54^.

### Flow analysis at the BA and BT

To determine the hemodynamic environment at the BT and BA, we performed CFD simulations. In brief, the 3D geometries from angiography were segmented using Vascular Modeling Toolkit (www.vmtk.org), then cropped and refined to include just the BA, BT, and bifurcating vessels. The segmented models were converted to volumetric meshes using ICEM-CFD (ANSYS, Inc.) and the flow-governing Navier–Stokes equations were solved using an open-source software, OpenFOAM (www.openfoam.org) under steady-state conditions. A uniform inlet boundary condition at the BA was defined using the time-averaged velocity obtained by TCD, and the outlet boundary conditions were assumed to split by obeying Murray’s law. For each rabbit, two CFD simulations were performed on the baseline geometry, using the initial and the post-surgery flow rates. Post-processing was performed in Paraview to obtain average WSS; at the BT bifurcation zone, the average was taken over the surface within a 1 mm diameter sphere centered at the apex of the bifurcation, and in the BA, WSS was averaged over the entire vessel segment.

### Tissue collection and laser microdissection

Immediately following euthanasia, animals were perfused with 1 U/mL heparin in 0.9% saline solution for 10 min. Then, the brain was removed, and the most posterior cerebral arteries, from the vertebral arteries to the posterior cerebral arteries and superior cerebellar arteries, were surgically excised. The BA with the BT and short portions of the downstream branches was placed in optimal cutting temperature compound and flash frozen in liquid nitrogen. Longitudinal sections of the BA and coronal sections through the BT were cut at 8 µm thickness, adhered to nuclease-free Foil Membrane Slides for use with the Leica laser microdissection system and stored at − 80 °C until laser microdissection. Immediately prior to microdissection, sections were fixed for 2 min with ice-cold 70% ethanol, washed twice for 60 s with ice-cold RNase-free water to remove residual embedding media, and dehydrated by one 2-min wash in 95% ethanol and two 2-min washes in 100% ethanol, then air dried for 1 h in a vacuum desiccator at room temperature. Dehydrated sections were micro-dissected on a Leica LMD6000 system (Leica Microsystems): ~ 10 μm wide strips were cut from the intimal layer (containing ECs) and separate strips were cut through the full thickness of the remaining medial layer (containing VSMCs). Intimal and medial tissue fragments from the BA and from a region extending ~ 100 µm distally from the apex of the BT were collected separately into dry microfuge tubes and held at − 80 °C until RNA extraction. To increase RNA yield, tissue from ~ 10 sections was combined for each sample.

### RNA extraction and sequencing

Total RNA was extracted and amplified from each sample tube using SMART-Seq v4 Ultra Low Input RNA Kits (Takara Bio). To check the quality of extraction and amplification, an aliquot of the end product was examined by electrophoresis using an Agilent 2100 BioAnalyzer RNA 6000 Pico Chip (Agilent, Las Vegas, NV). RNA samples to be sequenced had concentrations of 3.5–40 ng/µl and a broad molecular-weight distribution spanning 100–6000 bp and peaking at 800–2000 bp. RNA quality and sequencing metrics for each sample are provided in Supplemental Table 4.

cDNA libraries were prepared using Nextera DNA Library Preparation Kits (Illumina, San Diego, CA) and subjected to 75-cycle, single-read sequencing on an Illumina NextSeq500. For all data, per-cycle base-call (BCL) files generated by the NextSeq were converted to per-read FASTQ files using bcl2fastq v.2.20.0.422 using default parameters. The quality of the sequencing was reviewed using FastQC v.0.11.5. Detection of potential contamination was done using FastQ Screen v.0.11.1. FastQC and FastQ Screen quality reports were summarized using MultiQC v.1.5. No adapter sequences were detected, so no trimming was performed. Sequencing reads were aligned to rabbit genome, oryCun2, using STAR aligner^55^ Only uniquely mapped reads were retained for downstream analysis. Read counts were measured according to Ensembl gene annotation. Sequence alignments were compressed and sorted into binary alignment map (BAM) files using samtools v.1.9. Counting of mapped reads for genomic features was performed using Subread featureCounts v.1.6.2 using the parameters -s 2 –g gene_id –t exon –Q 60 -C, the annotation file specified with the -a parameter was the Ensembl reference OryCun2.0 (GCA_000003625.1). Aggregate quality control data (i.e. alignment statistics and feature assignment statistics) were again summarized using MultiQC.

### Gene expression analysis

We visualized how well intimal and medial samples separated at the two locations (BA, BT) using principal component analysis. Genes with a count sum > 0 across all samples were log transformed to use as input. Principal component analysis was created using the prcomp function in R. We then performed differential expression analysis using RPKM data from RNA-seq to investigate the effect of increased flow as induced by ligation on expression in the intima and media. The Bioconductor package edgeR (version 3.30.3)^56,57^ was used to perform four differential expression analyses: normal versus high flow in the intima at a bifurcation (BT), in the intima in a straight vessel (BA), in the media at a bifurcation (BT), and in the media in a straight vessel (BA). After estimating dispersion, edgeR identified DEGs by using a negative binomial distribution with generalized linear models and a quasi-likelihood F-test to identify DEGs^56,57^. Genes with maximum average expression > 1 RPKM in each group were used as input. Multiple hypothesis testing correction was performed using Benjamini–Hochberg false discovery rate correction. A gene was considered to be differentially expressed if the false discovery rate corrected p-value (q-value) < 0.05.

### Expression correlation analysis

To predict potential signals between intima and media, we looked for correlations between the expression patterns of intimal and medial genes. All genes with maximum expression > 1 RPKM and a q-value < 0.05 in any pair of comparisons within endothelial samples or within smooth muscle samples were included in the correlation analysis. For every possible intima-media gene pair, a Pearson correlation coefficient was calculated in R based on all of the available expression values (i.e., from the BA and BT of two ligated and two unligated animals for a total of 8 independent intimal measurements and eight matched medial measurement). Gene pairs with absolute correlation coefficient > 0.9 and p-value < 0.0001 were considered to have correlated expression.

### Gene ontology enrichment analysis

To examine the biological processes associated with different flow-induced patterns of gene expression in the intima, we separated DEGs into those with increased expression and those with decreased expression, in response to ligation-induced flow increase. Each set of DEGs was analyzed via the g:GOSt application of gProfiler^58^ to identify ontologies that were significantly overrepresented in each gene set (q-value < 0.05).

To characterize gene ontology enrichment among medial genes whose expression correlated with the expression of flow-sensitive intimal genes, it was necessary to first consider whether a medial gene correlated with an intimal gene that was up-regulated by increased flow or down-regulated by increased flow, and then to further separate medial genes that were positively correlated with the intimal gene’s expression from those that were negatively correlated. Thus, for each intimal gene of interest, all the positively correlating medial genes were input to identify one set of significantly enriched ontologies, and enriched ontologies for negatively correlating genes were determined separately.

In order to integrate the gene ontology information across all the different sets of medial genes that correlated with the many different flow-sensitive intimal genes, all the significant ontologies returned by g:Profiler for a given analysis (e.g., the positive medial correlates of intimal genes that increased in response to ligation) were pooled and ranked by occurrence. Terms that occurred in > 50% of collective ontologies were then summarized using the REduce and VIsualize Gene Ontology (REVIGO) tool^59^ to map relationships between ontologies and the associated rankings, with a semantic similarity cutoff of C = 0.90.

## Supplementary Information

Supplementary Information.

## Data Availability

Raw RNA-seq data files and tables of processed gene expression levels for all 16 samples from the n = 4 rabbits described in this publication can be found at NCBI’s GEO (accession number GSE169276).
