# Supplementary material for: Identification of intima-to-media signals for flow-induced vascular remodeling using correlative gene expression analysis
Source: Sci Rep. 2021 Aug 9;11:16142. doi: 10.1038/s41598-021-95403-x (PMC8352890; doi:10.1038/s41598-021-95403-x)
Supplement: Supplementary file 1 — Supplementary Information. [file 41598_2021_95403_MOESM1_ESM.pdf]

Supplemental Information for: Identification of intima-to-media signals for flow-induced vascular remodeling using correlative gene expression analysis

*John Kolega PhD[1,2], Kerry E. Poppenberg PhD[1,3], Hee-Woong Lim PhD[4], Liza C. Gutierrez MS[1,3], Sricharan S. Veeturi MS[1,5], Adnan H. Siddiqui MD-PhD[1,3], Hamidreza Rajabzadeh-Oghaz PhD[1,3], Vincent M. Tutino PhD[1,2,3,5]

[1]Canon Stroke and Vascular Research Center; [2]Department of Pathology and Anatomical Sciences, [3]Department of Neurosurgery, [5]Department of Mechanical and Aerospace Engineering, University at Buffalo, Buffalo, NY, USA
[4] Division of Biomedical Informatics, Cincinnati Children's Hospital Medical Center, Department of Pediatrics, University of Cincinnati College of Medicine, Cincinnati, OH, USA

***Corresponding Author:**
John Kolega, PhD
Department of Pathology & Anatomical Sciences
955 Main Street, Room 4102
Buffalo, NY 14203
E-mail: kolega@buffalo.edu
Phone: (716) 829-3527
Fax: (716) 829-2725

Supplemental Tables

Supplemental Table 1: Significantly enriched biological processes in gene ontologies of intimal DEGs.*

GO Name	GO ID	q-value
**Straight Vessel (BA) Intima**		
DEGs with increased expression		
regulation of hormone metabolic process	GO:0032350	0.008044
positive regulation of hydrogen peroxide biosynthetic process	GO:0010729	0.013828
regulation of thyroid hormone generation	GO:2000609	0.02073
secretion by cell	GO:0032940	0.026521
positive regulation of hydrogen peroxide metabolic process	GO:0010726	0.029006
export from cell	GO:0140352	0.035052
regulation of hydrogen peroxide biosynthetic process	GO:0010728	0.04967
DEGs with decreased expression		
keratan sulfate catabolic process	GO:0042340	0.018566
**Bifurcation (BT) Intima**		
DEGs with increased expression		
regulation of hormone metabolic process	GO:0032350	0.006677353
regulation of cell motility	GO:2000145	0.01564662
locomotion	GO:0040011	0.017394735
localization of cell	GO:0051674	0.018853748
cell motility	GO:0048870	0.018853748
regulation of locomotion	GO:0040012	0.023637559
inflammatory response	GO:0006954	0.029681431
regulation of body fluid levels	GO:0050878	0.030570609
regulation of cellular component movement	GO:0051270	0.033783587
negative regulation of coagulation	GO:0050819	0.03990575
regulation of cell migration	GO:0030334	0.047124802
DEGs with decreased expression		
regulation of hydrolase activity	GO:0051336	0.000197004
negative regulation of hydrolase activity	GO:0051346	0.001091982
negative regulation of endopeptidase activity	GO:0010951	0.00218846
negative regulation of peptidase activity	GO:0010466	0.003533473
regulation of endopeptidase activity	GO:0052548	0.012402965
regulation of cell population proliferation	GO:0042127	0.025377696
regulation of peptidase activity	GO:0052547	0.026346162
regulation of leukocyte chemotaxis	GO:0002688	0.031288446
regulation of chemotaxis	GO:0050920	0.038111509

***** (Abbreviations: DEG=differentially expressed gene, BA=basilar artery, BT=basilar terminus, GO=gene ontology)

Supplemental Table 2: Significantly enriched biological processes in gene ontologies of medial DEGs.*

GO Name	GO ID	q-value
**<u>Straight Vessel (BA) Media</u>**		
DEGs with increased expression		
positive regulation of protein localization to chromosome, telomeric region	GO:1904816	3.29E-08
regulation of protein localization to chromosome, telomeric region	GO:1904814	1.21E-07
positive regulation of establishment of protein localization to telomere	GO:1904851	4.21E-07
regulation of establishment of protein localization to telomere	GO:0070203	9.19E-07
regulation of protein localization to Cajal body	GO:1904869	9.19E-07
positive regulation of protein localization to Cajal body	GO:1904871	9.19E-07
protein localization to Cajal body	GO:1904867	1.82E-06
regulation of establishment of protein localization to chromosome	GO:0070202	1.82E-06
protein localization to nuclear body	GO:1903405	1.82E-06
protein localization to nucleoplasm	GO:1990173	5.83E-06
positive regulation of telomerase RNA localization to Cajal body	GO:1904874	9.63E-06
anatomical structure morphogenesis	GO:0009653	1.59E-05
response to organic substance	GO:0010033	3.36E-05
establishment of protein localization to telomere	GO:0070200	3.48E-05
regulation of telomerase RNA localization to Cajal body	GO:1904872	3.48E-05
protein localization to chromosome, telomeric region	GO:0070198	4.33E-05
telomerase RNA localization to Cajal body	GO:0090671	5.05E-05
RNA localization to nucleus	GO:0090685	5.05E-05
telomerase RNA localization	GO:0090672	5.05E-05
RNA localization to Cajal body	GO:0090670	5.05E-05
positive regulation of protein localization to nucleus	GO:1900182	5.72E-05
wound healing	GO:0042060	8.94E-05
circulatory system development	GO:0072359	0.000352
regulation of localization	GO:0032879	0.000439
establishment of protein localization to chromosome	GO:0070199	0.000515
regulation of cell migration	GO:0030334	0.000753
positive regulation of cell migration	GO:0030335	0.000838
cellular response to organic substance	GO:0071310	0.000904
protein folding	GO:0006457	0.000972
positive regulation of cellular protein localization	GO:1903829	0.00109
positive regulation of cell motility	GO:2000147	0.001538
regulation of protein localization	GO:0032880	0.001644
positive regulation of cellular component movement	GO:0051272	0.002171
positive regulation of telomere maintenance via telomerase	GO:0032212	0.002208
positive regulation of locomotion	GO:0040017	0.002287
blood vessel morphogenesis	GO:0048514	0.00229
regulation of telomere maintenance via telomerase	GO:0032210	0.0024

regulation of cell motility	GO:2000145	0.002431
response to wounding	GO:0009611	0.00319
positive regulation of telomere maintenance via telomere lengthening	GO:1904358	0.003722
regulation of protein localization to nucleus	GO:1900180	0.004054
blood vessel development	GO:0001568	0.004556
cellular response to chemical stimulus	GO:0070887	0.0048
regulation of locomotion	GO:0040012	0.00522
positive regulation of cellular component organization	GO:0051130	0.005307
regulation of anatomical structure morphogenesis	GO:0022603	0.005841
tissue remodeling	GO:0048771	0.006721
tissue development	GO:0009888	0.00686
regulation of telomere maintenance via telomere lengthening	GO:1904356	0.006947
muscle structure development	GO:0061061	0.009228
toxin transport	GO:1901998	0.009284
regulation of establishment of protein localization	GO:0070201	0.009445
vasculature development	GO:0001944	0.009805
regulation of cellular component movement	GO:0051270	0.010045
binding of sperm to zona pellucida	GO:0007339	0.010661
angiogenesis	GO:0001525	0.011714
telomere maintenance via telomerase	GO:0007004	0.012876
response to cytokine	GO:0034097	0.014255
vascular endothelial growth factor receptor signaling pathway	GO:0048010	0.015341
RNA-dependent DNA biosynthetic process	GO:0006278	0.015606
export from cell	GO:0140352	0.016198
anatomical structure homeostasis	GO:0060249	0.01742
secretion by cell	GO:0032940	0.019721
secretion	GO:0046903	0.021386
protein localization to nucleus	GO:0034504	0.022813
response to stress	GO:0006950	0.024796
positive regulation of telomere maintenance	GO:0032206	0.025659
extracellular matrix organization	GO:0030198	0.028139
extracellular structure organization	GO:0043062	0.028982
myeloid leukocyte activation	GO:0002274	0.033009
regulation of epithelial cell migration	GO:0010632	0.033525
tube morphogenesis	GO:0035239	0.034179
anatomical structure development	GO:0048856	0.037278
cell-cell recognition	GO:0009988	0.037498
positive regulation of epithelial cell migration	GO:0010634	0.039893
tube development	GO:0035295	0.040527
telomere maintenance via telomere lengthening	GO:0010833	0.040653
regulation of telomere maintenance	GO:0032204	0.040653
integrin-mediated signaling pathway	GO:0007229	0.040691
regulation of DNA biosynthetic process	GO:2000278	0.040691
tissue morphogenesis	GO:0048729	0.045289
regulation of cellular localization	GO:0060341	0.046172
DEGs with decreased expression		
extracellular matrix organization	GO:0030198	1.44E-07

extracellular structure organization	GO:0043062	1.51E-07
collagen fibril organization	GO:0030199	0.000274
cell adhesion	GO:0007155	0.01331
biological adhesion	GO:0022610	0.014502
cell-substrate adhesion	GO:0031589	0.027067
system development	GO:0048731	0.031141
cellular component organization	GO:0016043	0.039335
regulation of multicellular organismal process	GO:0051239	0.040545

Bifurcation (BT) Media

DEGs with increased expression

secretion by cell	GO:0032940	8.98E-08
regulated exocytosis	GO:0045055	1.92E-07
export from cell	GO:0140352	2.72E-07
secretion	GO:0046903	3.84E-07
exocytosis	GO:0006887	1.04E-06
wound healing	GO:0042060	2.45E-06
neutrophil mediated immunity	GO:0002446	1.27E-05
response to wounding	GO:0009611	2.3E-05
myeloid leukocyte mediated immunity	GO:0002444	7.96E-05
immune effector process	GO:0002252	0.000204
neutrophil degranulation	GO:0043312	0.000245
neutrophil activation involved in immune response	GO:0002283	0.000269
response to organic substance	GO:0010033	0.000342
neutrophil activation	GO:0042119	0.000397
regulation of coagulation	GO:0050818	0.000411
establishment of localization in cell	GO:0051649	0.000443
cell activation	GO:0001775	0.000465
granulocyte activation	GO:0036230	0.000487
negative regulation of coagulation	GO:0050819	0.000808
leukocyte activation involved in immune response	GO:0002366	0.001157
leukocyte degranulation	GO:0043299	0.00119
cell activation involved in immune response	GO:0002263	0.001266
myeloid cell activation involved in immune response	GO:0002275	0.001551
cellular localization	GO:0051641	0.002287
response to chemical	GO:0042221	0.004167
transport	GO:0006810	0.004324
localization	GO:0051179	0.005
vesicle-mediated transport	GO:0016192	0.005808
myeloid leukocyte activation	GO:0002274	0.006879
tissue remodeling	GO:0048771	0.007778
immune system process	GO:0002376	0.00834
leukocyte mediated immunity	GO:0002443	0.009673
leukocyte activation	GO:0045321	0.011766
establishment of localization	GO:0051234	0.011994
positive regulation of cell migration	GO:0030335	0.013711
blood coagulation	GO:0007596	0.014094
hemostasis	GO:0007599	0.016434
coagulation	GO:0050817	0.017997
positive regulation of cell motility	GO:2000147	0.022315
regulation of response to wounding	GO:1903034	0.022571

response to stimulus	GO:0050896	0.023561
positive regulation of supramolecular fiber organization	GO:1902905	0.027681
positive regulation of cellular component movement	GO:0051272	0.029426
positive regulation of locomotion	GO:0040017	0.030684
regulation of localization	GO:0032879	0.031315
regulation of wound healing	GO:0061041	0.033545
regulation of blood coagulation	GO:0030193	0.040146
regulation of hemostasis	GO:1900046	0.04373
regulation of supramolecular fiber organization	GO:1902903	0.044816
DEGs with decreased expression		
extracellular matrix organization	GO:0030198	0.001966
extracellular structure organization	GO:0043062	0.002025
cell-substrate adhesion	GO:0031589	0.004516
muscle structure development	GO:0061061	0.011581
muscle tissue development	GO:0060537	0.017933
regulation of hydrolase activity	GO:0051336	0.036424
actin filament-based process	GO:0030029	0.044306

***** (Abbreviations: DEG=differentially expressed gene, BA=basilar artery, BT=basilar terminus, GO=gene ontology)

Supplemental Table 3. Correlations between expression of intimal and medial genes.*

Intimal gene	#medial corr.	Predicted location of gene product	Type
ANKRD1	301	Cytoplasm	Transcription regulator
PLS3	296	Cytoplasm	Other
MAP2K3	292	Cytoplasm	Kinase
TBC1D4	292	Cytoplasm	Other
ENPP2	290	Plasma Membrane	Enzyme
ATP5PF	290	Cytoplasm	Transporter
DPEP2	288	Plasma Membrane	Peptidase
NPY1R	287	Plasma Membrane	G protein-coupled receptor
SERPINA6	287	Extracellular Space	Other
DDX24	285	Nucleus	Enzyme
SDF2L1	284	Cytoplasm	Other
CYTL1	283	Extracellular Space	Cytokine
CLIC5	283	Cytoplasm	Ion channel
ADGRL4	282	Plasma Membrane	G protein-coupled receptor
ABCC9	282	Plasma Membrane	Ion channel
TM4SF18	282	Other	Other
NDP	281	Extracellular Space	Growth factor
ALDH2	273	Cytoplasm	Enzyme
FOLR2	272	Plasma Membrane	Transporter
MSN	271	Plasma Membrane	Other
ITGB3	271	Plasma Membrane	Transmembrane receptor
SRXN1	270	Cytoplasm	Enzyme
ARPC2	270	Cytoplasm	Other
SPP1	269	Extracellular Space	Cytokine
C1QTNF7	267	Extracellular Space	Other
SORT1	266	Plasma Membrane	G protein-coupled receptor
RFTN1	262	Plasma Membrane	Other
BEX3	261	Cytoplasm	Other
GLIPR1	257	Extracellular Space	Other
YBX3	257	Nucleus	Transcription regulator
GPRC5A	256	Plasma Membrane	G protein-coupled receptor
ST6GALNAC4	255	Cytoplasm	Enzyme
MT3	255	Cytoplasm	Other
SELENBP1	255	Cytoplasm	Other
SERPINE1	255	Extracellular Space	Other
PTPN13	255	Cytoplasm	Phosphatase
PLA2R1	255	Plasma Membrane	Transmembrane receptor
DUOXA2	254	Cytoplasm	Other
LYPD6	254	Extracellular Space	Other
CALCRL	252	Plasma Membrane	G protein-coupled receptor
GNG11	252	Plasma Membrane	Other
LUM	251	Extracellular Space	Other
CYR61	250	Extracellular Space	Other
WASF3	244	Cytoplasm	Other
UPF3A	244	Nucleus	Transporter
ALDH6A1	243	Cytoplasm	Enzyme
ABI3BP	239	Extracellular Space	Other

ADAM15	235	Plasma Membrane	Peptidase
ADAMTS9	235	Extracellular Space	Peptidase
GJB3	234	Plasma Membrane	Transporter
CST6	233	Extracellular Space	Other
ELOVL1	229	Cytoplasm	Enzyme
HYAL2	229	Cytoplasm	Enzyme
TIMP1	224	Extracellular Space	Cytokine
C9orf3	223	Cytoplasm	Peptidase
TGM2	222	Cytoplasm	Enzyme
LRRC8C	221	Plasma Membrane	Ion channel
IL15	219	Extracellular Space	Cytokine
QSOX1	216	Cytoplasm	Enzyme
CRABP1	216	Cytoplasm	Transporter
PLA2G7	215	Extracellular Space	Enzyme
NT5C3A	215	Cytoplasm	Phosphatase
MALL	214	Plasma Membrane	Other
TACR1	213	Plasma Membrane	G protein-coupled receptor
TSPAN1	206	Cytoplasm	Other
SLC7A7	206	Plasma Membrane	Transporter
DBNDD2	205	Cytoplasm	Other
DUOXA1	205	Plasma Membrane	Other
OMD	204	Extracellular Space	Other
MFAP5	202	Extracellular Space	Other
RDH10	201	Nucleus	Enzyme
OGN	197	Extracellular Space	Growth factor
SLC22A1	195	Plasma Membrane	Transporter
TNNC1	192	Cytoplasm	Other
LDHA	185	Cytoplasm	Enzyme
RYR2	185	Plasma Membrane	Ion channel
SEMA3F	185	Extracellular Space	Other
P2RY2	182	Plasma Membrane	G protein-coupled receptor
ASPA	180	Cytoplasm	Enzyme
FABP5	179	Cytoplasm	Transporter
RENBP	178	Cytoplasm	Enzyme
NR1D2	178	Nucleus	ligand-dependent nuclear receptor
SELENOP	178	Extracellular Space	Other
PLAUR	178	Plasma Membrane	Transmembrane receptor
PLPP1	174	Plasma Membrane	Phosphatase
PRDM6	174	Nucleus	Transcription regulator
HTR2A	171	Plasma Membrane	G protein-coupled receptor
TMTC4	171	Other	Other
MRC1	171	Plasma Membrane	Transmembrane receptor
LCAT	169	Extracellular Space	Enzyme
ENPP6	167	Cytoplasm	Enzyme
SYDE2	167	Cytoplasm	Other
CA3	166	Cytoplasm	Enzyme
DAB2	166	Plasma Membrane	Other
CD93	164	Plasma Membrane	Other
SLC13A4	164	Plasma Membrane	Transporter
RLA-DR-ALPHA	161	Plasma Membrane	Other

ARNTL2	158	Nucleus	Transcription regulator
IFI44L	157	Nucleus	Other
SLC6A13	155	Plasma Membrane	Transporter
HIPK1	154	Nucleus	Kinase
C2	152	Extracellular Space	Peptidase
ANGPTL4	148	Extracellular Space	Other
IGFBP2	148	Extracellular Space	Other
STEAP1	148	Plasma Membrane	Transporter
ADAMTS6	146	Extracellular Space	Peptidase
FNDC1	145	Plasma Membrane	Other
TDRP	144	Cytoplasm	Other
CCNYL1	141	Plasma Membrane	Other
CD74	140	Plasma Membrane	Transmembrane receptor
BTG2	139	Nucleus	Transcription regulator
ATP1B2	139	Plasma Membrane	Transporter
PROCR	136	Plasma Membrane	Other
TTR	128	Extracellular Space	Transporter
SAT1	127	Cytoplasm	Enzyme
S100A1	126	Cytoplasm	Other
LZTFL1	119	Cytoplasm	Other
COL23A1	113	Plasma Membrane	Other
MLLT10	111	Nucleus	Transcription regulator
ACSS3	109	Cytoplasm	Enzyme
CMBL	106	Cytoplasm	Enzyme
GDE1	106	Plasma Membrane	Enzyme
C1QC	103	Extracellular Space	Other
TYROBP	101	Plasma Membrane	Transmembrane receptor
MAPK4	99	Cytoplasm	Kinase
NTRK2	99	Plasma Membrane	Kinase
IGFBP6	99	Extracellular Space	Other
EPHX1	99	Cytoplasm	Peptidase
PLAT	99	Extracellular Space	Peptidase
SLC22A6	99	Plasma Membrane	Transporter
NR4A1	96	Nucleus	Ligand-dependent nuclear receptor
ZNF521	96	Nucleus	Transcription regulator
RARRES2	95	Plasma Membrane	Transmembrane receptor
JUN	93	Nucleus	Transcription regulator
HBEGF	92	Extracellular Space	Growth factor
COLEC12	92	Plasma Membrane	Transmembrane receptor
PTN	90	Extracellular Space	Growth factor
LPAR1	88	Plasma Membrane	G protein-coupled receptor
HP	86	Extracellular Space	Peptidase
SLC22A8	82	Plasma Membrane	Transporter
BEND5	80	Cytoplasm	Other
PROS1	78	Extracellular Space	Other
RARRES1	78	Plasma Membrane	Other
PDGFRL	77	Plasma Membrane	Kinase
C1QA	75	Extracellular Space	Other
POPDC3	75	Other	Other
ARHGEF26	73	Plasma Membrane	Other
DPP4	72	Plasma Membrane	Peptidase

IGDCC4	69	Plasma Membrane	Other
ENTPD3	68	Plasma Membrane	Enzyme
KBTBD4	68	Other	Other
SMARCA1	68	Nucleus	Transcription regulator
F3	68	Plasma Membrane	Transmembrane receptor
BMP2	66	Extracellular Space	Growth factor
COL14A1	66	Extracellular Space	Other
PXK	63	Cytoplasm	Kinase
LAPTM5	63	Plasma Membrane	Other
NID2	63	Extracellular Space	Other
SCD5	62	Cytoplasm	Enzyme
EPHA3	59	Plasma Membrane	Kinase
KCNC2	55	Plasma Membrane	Ion channel
ARHGAP11A	54	Cytoplasm	Other
SLC16A9	50	Other	Other
MAN1C1	49	Cytoplasm	Enzyme
ITIH5	42	Plasma Membrane	Other
PIP4P2	40	Cytoplasm	Phosphatase
SLC26A7	40	Plasma Membrane	Transporter
ITIH2	38	Extracellular Space	Other
SLC7A11	37	Plasma Membrane	Transporter
IGF1	36	Extracellular Space	Growth factor
FAM83D	35	Cytoplasm	Other
PLBD1	32	Cytoplasm	Enzyme
IFT74	32	Cytoplasm	Other
KRT19	32	Cytoplasm	Other
RSPO3	29	Extracellular Space	Kinase
CHI3L2	28	Extracellular Space	Enzyme
VIT	25	Extracellular Space	Other
EYA1	25	Nucleus	Phosphatase
ISG15	24	Extracellular Space	Other
BST1	21	Plasma Membrane	Enzyme
CXCL11	19	Extracellular Space	Cytokine
ZNF396	15	Nucleus	Transcription regulator
CACHD1	12	Other	Other
LRRC2	12	Other	Other
RERG	11	Nucleus	Enzyme
SERPINF1	11	Extracellular Space	Other
SEPSECS	10	Cytoplasm	Enzyme
BMP3	10	Extracellular Space	GF
ANXA8	8	Plasma Membrane	Other
IFIT2	7	Cytoplasm	Other
SORL1	7	Cytoplasm	Transporter
CXCL10	6	Extracellular Space	Cytokine
SAMD9L	6	Extracellular Space	Other
ATP1B1	6	Plasma Membrane	Transporter

***** (Abbreviations: Corr.=correlates)

Supplemental Table 4 Quality control metrics for RNA and sequencing.*

Sample ID	[RNA] ng/µl	Sequences x10^{6}	%GC	%Aligned
Rb3 BA m	3.81	38.1	44	81.5
Rb3 BT m	1.74	13.7	44	78.9
Rb3 BA i	9.16	57.1	44	84.5
Rb3 BT i	32.40	42.7	45	82.9
Rb4 BA m	37.90	20.7	47	82.6
Rb4 BT m	5.16	28.4	43	73.0
Rb4 BA i	18.88	46.2	46	82.5
Rb4 BT i	6.15	28.7	44	76.2
Rb5 BA m	37.70	5.9	45	86.3
Rb5 BT m	40.80	10.4	45	83.5
Rb5 BA i	1.44	32.6	43	81.5
Rb5 BT i	3.55	30.5	45	87.2
Rb6 BA m	15.55	11.1	46	87.8
Rb6 BT m	15.92	24.9	45	85.7
Rb6 BA i	26.47	14.3	46	88.0
Rb6 BT i	15.16	31.7	46	86.9

* (Abbreviations: Rb=rabbit number, BT = basilar terminus, BA = basilar artery, i= intima, m= media)

Supplemental Figures

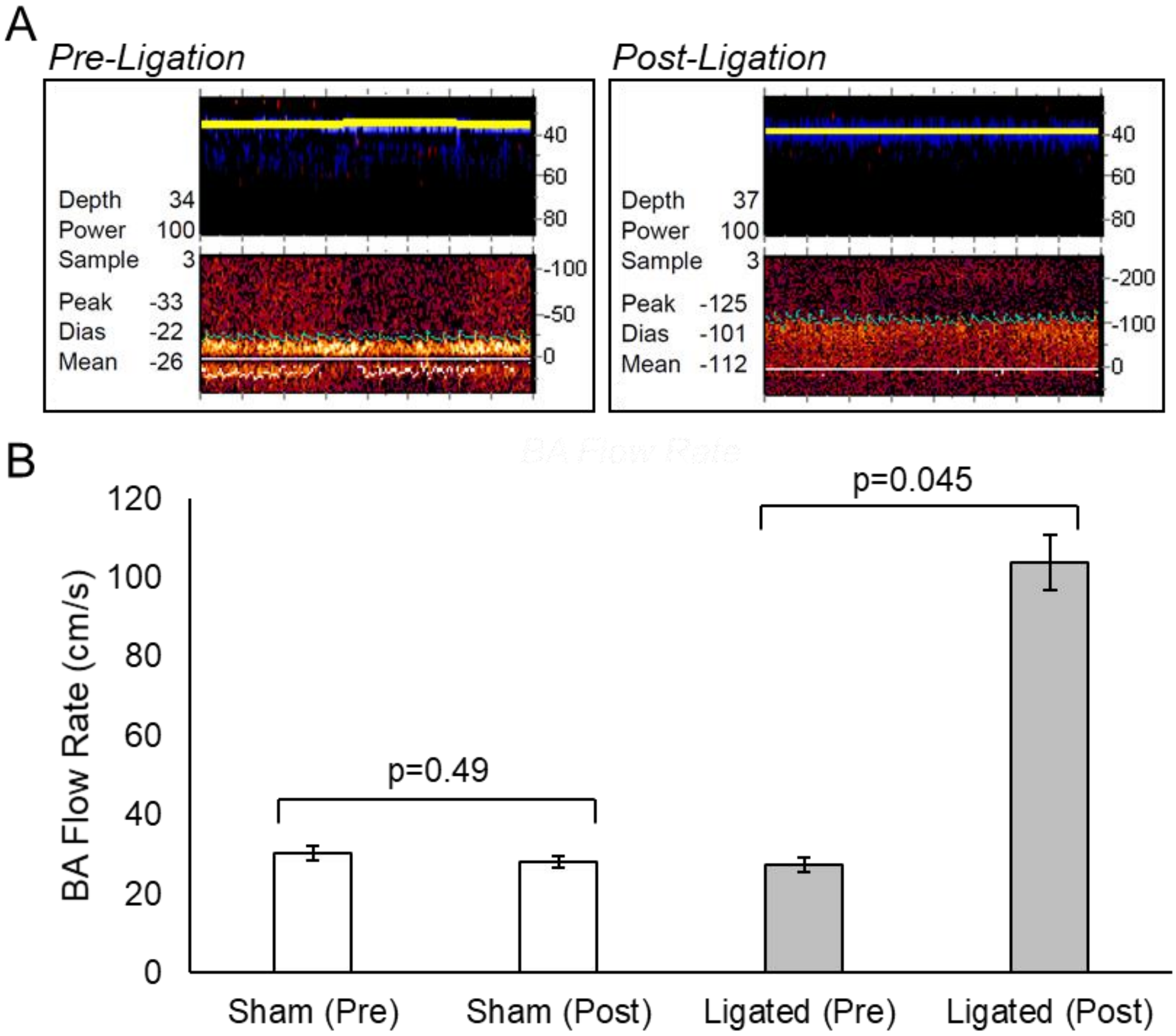

Supplemental Figure 1: Flow rate measurements. (**A**)**.** Example TCD data for one ligated rabbit. Across the ligated rabbits, the pre-ligation average velocity (v) = 27.5 cm/s, while post-ligation average v = 104.0 cm/s. In the shams, the pre-ligation average v = 29.4 cm/s, while post-ligation average v = 29.0 cm/s. (**B**)**.** In control animals, sham surgery did not change BA flow rate, whereas in experimental animals, bilateral CCA ligation significantly increased the BA flow rate.

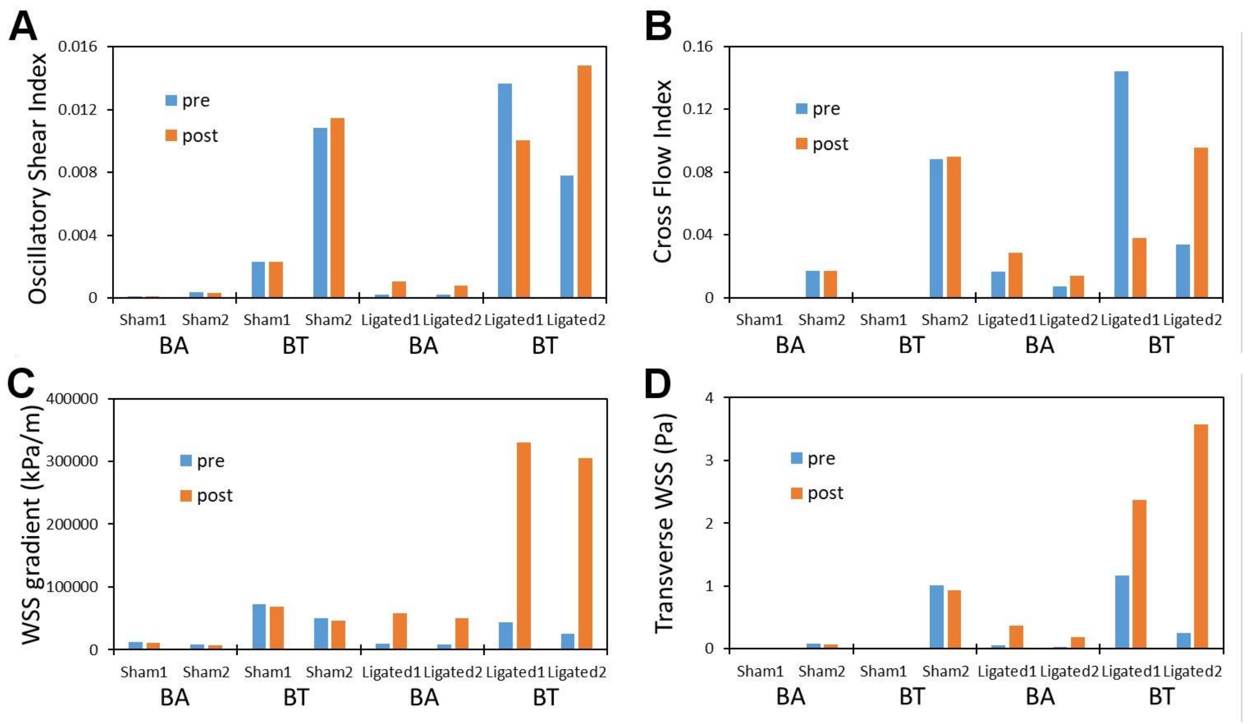

Supplemental Figure 2: Additional hemodynamic parameters. (**A**) Oscillating flow index, (**B**) cross flow index, (**C**) WSS gradient in the direction of flow and (**D**) transverse WSS were mapped over the 3D vascular geometry for each animal. Values for the BA were averaged over the length of the vessel, and values for the BT were averaged over the vessel surface within a 1 mm diameter sphere centered at the apex of the bifurcation (see Supplemental Figure 3 for regions that were averaged). Oscillating flow index (A) and cross flow index (B), were not appreciably affected by ligation, whereas WSS gradient in the direction of flow (C) and transverse WSS (D) were higher in ligated animal, especially at the BT.

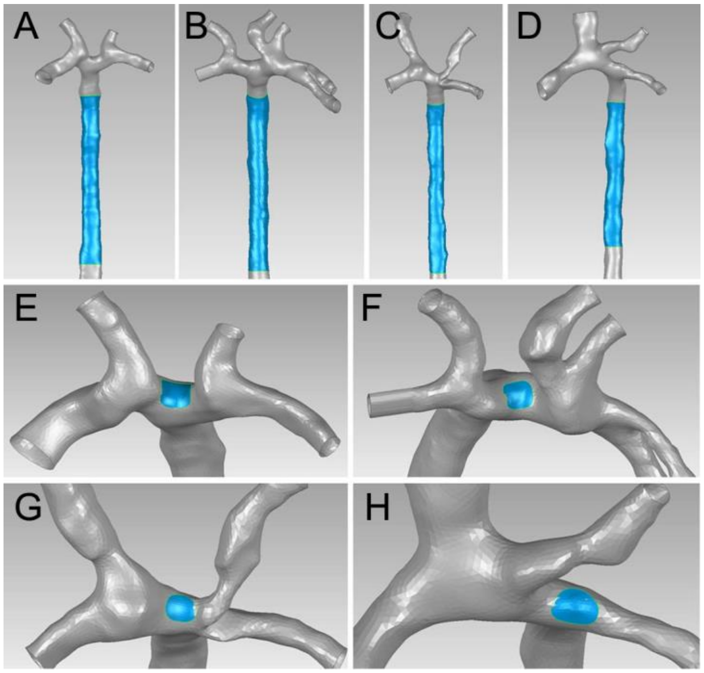

Supplemental Figure 3: Locations of WSS measurements and tissue collection. Panels A-D show the geometries of the 4 vasculatures that were analyzed in this study, with the region of the BA over which WSS was averaged marked in blue. Long longitudinal strips of intima and media were dissected throughout this same blue region when collecting tissue by laser microdissection for the RNAseq analyses. (**A**) Sham 1, (**B**) Sham 2, (**C**) Ligated 1, (**D**) Ligated 2. Panels E-H show a magnified view of the BT, with the region of which WSS was averaged and within intima and media tissue were collected again shaded in blue. (**E**) Sham 1, (**F**) Sham 2, (**G**) Ligated 1, (**H**) Ligated 2.

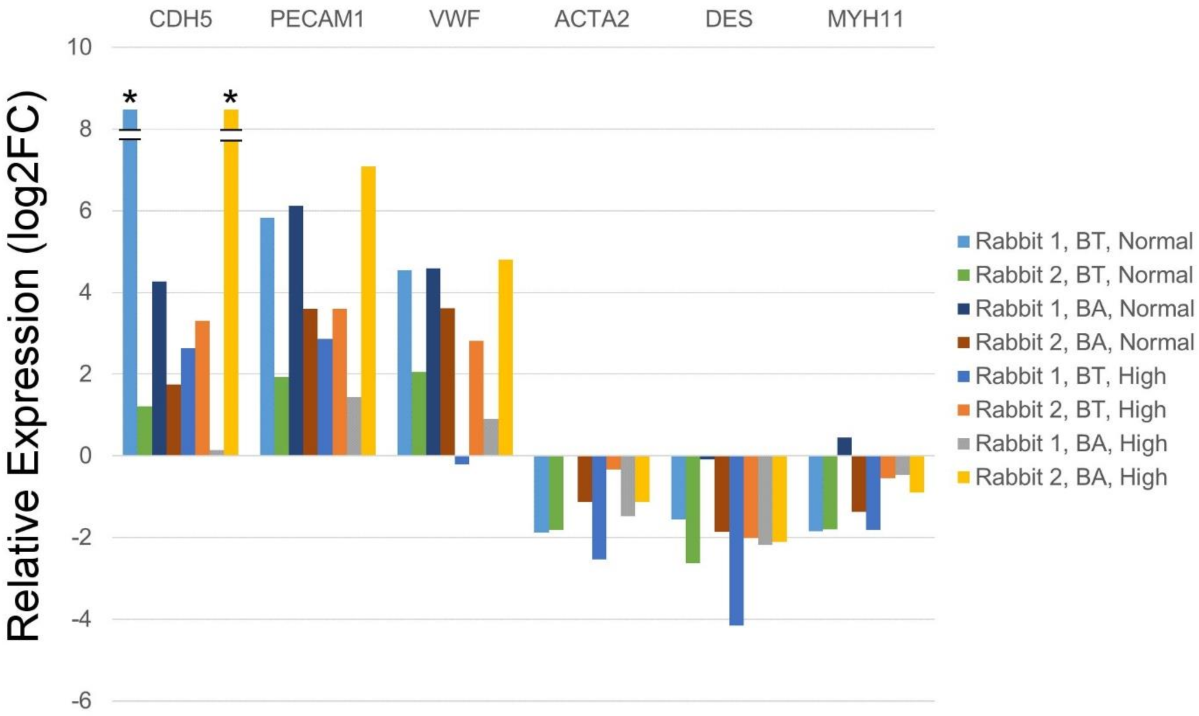

Supplemental Figure 4: Relative expression of endothelial and smooth-muscle markers in intimal and medial transcriptomes. To assess how well intimal and medial tissues were separated by microdissection, the expression of genes for markers of endothelial cells and smooth muscle were compared for each paired set of intimal and medial samples from the 8 sampling locations in the study. The log2 fold change between intimal and medial tissue are shown for the endothelial markers, *PECAM1*, *VWF*, and *CDH*5, on the left, and the smooth-muscle markers, *DES*, *ACTA2*, and *MYH1*, on the right. * marks measurements for which there was no detectable expression in the medial samples, so the fold change would be mathematically infinite. Endothelial markers are much more abundant in intimal samples, and smooth-muscle markers are less abundant than in their intimal counterparts

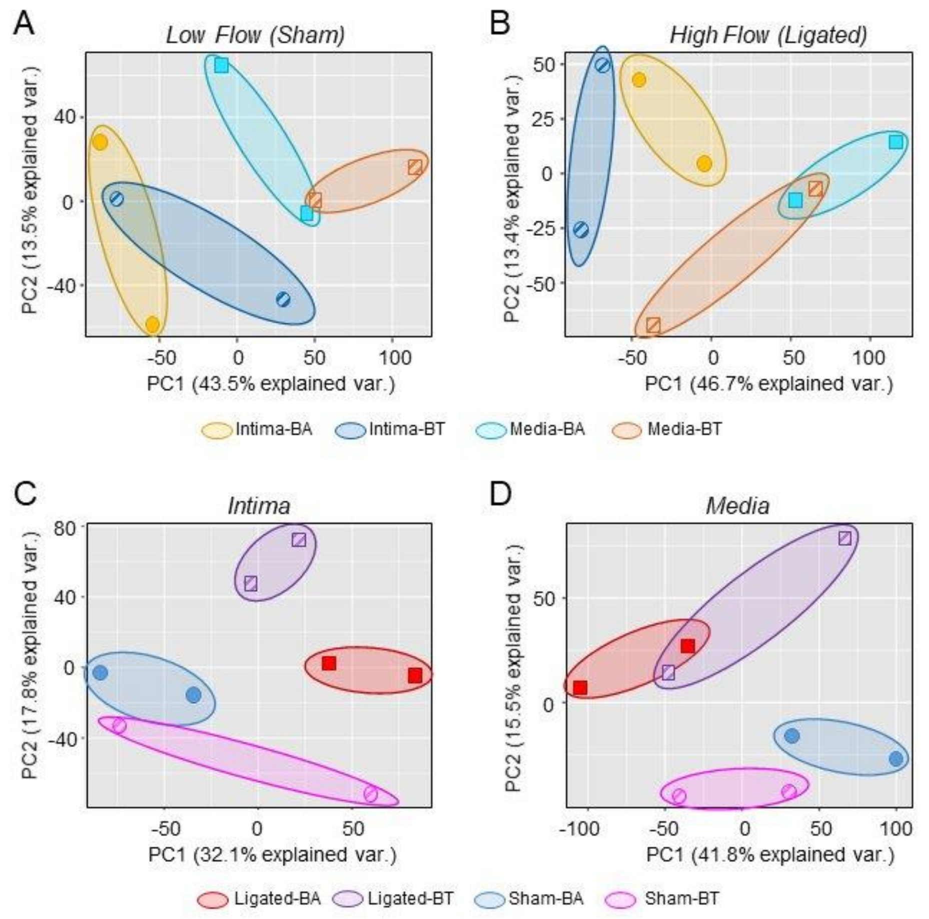

Supplemental Figure 5: Additional Principal Component Analysis of gene expression. Principal component analysis was performed on the gene expression levels for all genes that had a count sum >0 across all samples. The first 2 principal components for the collective gene expression in each sample are plotted for samples from **(A)** all sham rabbits, **(B)** all ligated rabbits, **(C)** all intimal samples, and **(D)** all medial samples. Colored ellipses indicate paired duplicates from 2 different rabbits. Intimal and medial expression separated well in both sham and ligated animals (A and B), while samples from different locations tended to be less clearly separated and displayed more overlap; e.g., intima-BA and intima-BT in A). Panels C and D illustrate the effect of increased flow, with the clearest separations occurring between ligated versus sham samples for both intima and media genes. Samples were again less clearly distinguished by location (e.g., ligated-BA vs ligated–BT in D).
